# Should ice application be replaced with neurocryostimulation for the treatment of acute lateral ankle sprains? A randomized clinical trial

**DOI:** 10.1186/s13047-020-00436-6

**Published:** 2020-12-01

**Authors:** Jean Tittley, Luc J. Hébert, Jean-Sébastien Roy

**Affiliations:** 1 Department of Rehabilitation, Faculty of Medicine, Université Laval, Quebec City, Quebec Canada; 2 Center for Interdisciplinary Research in Rehabilitation and Social Integration, Quebec Rehabilitation Institute, Quebec City, Quebec Canada; 3 Department of Radiology and Nuclear Medicine, Faculty of Medicine, Université Laval, Quebec City, Quebec Canada

**Keywords:** Lateral ankle sprain, Neurocryostimulation, Cryotherapy, Rehabilitation

## Abstract

**Study design:**

Single-blind parallel group randomized clinical trial.

**Objectives:**

To compare the effects of neurocryostimulation (NCS) with those of traditional ice application on functional recovery, pain, edema and ankle dorsiflexion range of motion (ROM) in individuals receiving physiotherapy treatments for acute lateral ankle sprains (LAS).

**Background:**

Ankle sprain is a very common injury and its management is often costly, with important short- and long-term impacts on individuals and society. As new methods of therapy using cold (cryotherapy) are emerging for the treatment of musculoskeletal conditions, little evidence exists to support their use. NCS, which provokes a rapid cooling of the skin with the liberation of pressured CO^2^, is a method believed to accelerate the resorption of edema and recovery in the case of traumatic injuries.

**Methods:**

Forty-one participants with acute LAS were randomly assigned either to a group that received in-clinic physiotherapy treatments and NCS (experimental NCS group, *n* = 20), or to a group that received the same in-clinic physiotherapy treatments and traditional ice application (comparison ice group, *n* = 21). Primary (Lower Extremity Functional Scale - LEFS) and secondary (visual analog scale for pain intensity at rest and during usual activities in the last 48 h, Figure of Eight measurement of edema, and weight bearing lunge for ankle dorsiflexion range of motion) outcomes were evaluated at baseline (T0), after one week (T1), two weeks (T2), four weeks (T4) and finally, after six weeks (T6). The effects of interventions were assessed using two-way ANOVA-type Nonparametric Analysis for Longitudinal Data (nparLD).

**Results:**

No significant group-time interaction or group effect was observed for all outcomes (0.995 ≥ *p* ≥ 0.057) following the intervention. Large time effects were however observed for all outcomes (*p* <  0.0001).

**Conclusion:**

Results suggest that neurocryostimulation is no more effective than traditional ice application in improving functional recovery, pain, edema, and ankle dorsiflexion ROM during the first six weeks of physiotherapy treatments in individuals with acute LAS.

**Level of evidence:**

Therapy, level 1b.

**Trial registration:**

ClinicalTrials.gov, NCT02945618. Registered 23 October 2016 - Retrospectively registered (25 participants recruited prior to registration, 17 participants after).

## Background

Ankle sprains represent up to 25% of all musculoskeletal injuries, and half of them are sports-related [1]. Sprain can be defined as the injury that occurs when a tensile force causes stretching, partial tearing or complete tearing of a ligament [2]. Lateral sprains are the most frequent type of ankle sprain, occurring at least two times more frequently than all other ankle sprains combined [3]. Considering that 20 to 50% of individuals who suffer ankle sprains develop chronic pain or chronic ankle instability, the prognosis is less than optimistic [4]. These realities impel constant efforts to identify optimal treatment plans.

The most widely-used treatment for acute-phase ankle sprains is summarized by the RICE acronym (Rest, Ice, Compression, Elevation) [5]. However, the rationale behind the use of RICE, as well as for each of its individual components, is largely based on a very limited evidence, consisting mainly of low-quality clinical trials and laboratory studies on healthy subjects or on animals [6–8]. Systematic reviews on acute ankle sprain treatments report evidence supporting the use of early mobilization (weight-bearing as tolerated and mobilization exercises), analgesic drugs, splints or braces, as well as manual therapy [3, 8–11]. However, scientific evidence on the effect of cryotherapy specifically is limited and inconsistent. That said, one systematic review concluded that cryotherapy is effective in decreasing pain in the short term (over a one-week period) in acute-phase of soft tissue injuries [12]. In addition, a 2018 clinical guideline reports that there is no evidence that cryotherapy alone is effective in improving pain, function, or swelling in acute lateral ankle sprains (LAS), but that it improves ankle function in the short term when combined with exercises [8]. Moreover, no consensus has been reached concerning the best cryotherapy protocols or application methods [12, 13].

Neurocryostimulation (NCS), also known as gaseous hyperbaric cryotherapy, is a treatment modality in which compressed carbonic gas is projected from a medical gun onto a patient’s skin at high speed. A rapid and considerable drop in skin temperature ensues, causing thermal shock, which, in healthy subjects, is a swift systemic response resulting in cutaneous vasoconstriction and increased blood pressure [14, 15]. These effects on the autonomic nervous system have been proposed to accelerate edema resorption, thus quickening recovery and healing following soft tissue injuries [16]. Other suggested effects of NCS include analgesia (by nociceptor inhibition), decreased inflammation (by suppression of enzyme activity), vasomotor effects (by profound vasodilation after 20 to 30 s of application), and muscle relaxation (resulting from a myostatic reflex in the medulla) [16]. However, these proposed effects remain highly speculative, as only few studies have evaluated the effect of NCS in symptomatic populations, and most of them present important methodological shortcomings in either the experimental design (no comparison group) [16] or the NCS application protocol (attaining suboptimal temperatures) [17].

The objective of this study was to evaluate the effects of neurocryostimulation (NCS) in comparison with those of traditional ice application on functional recovery, pain, edema and ankle dorsiflexion range of motion (ROM) in individuals receiving physiotherapy treatments for acute LAS, using a single-blind randomized clinical trial. The hypothesis was that participants receiving NCS treatment would improve more rapidly for all measured variables than those treated with ice. This RCT was registered on ClinicalTrials.gov (NCT02945618).

## Methods

### Participants

Potential participants were recruited using a university community’s electronic mailing list (*Université Laval*, Quebec City) and at the university’s physiotherapy clinic. A physiotherapist in charge of recruitment and evaluations contacted all interested individuals for pre-screening and scheduled a first appointment at the clinic with potential participants to confirm the following criteria: 1) had experienced a moderate to high grade LAS in the 72 h preceding recruitment, 2) were aged 18 and above, and 3) were available to participate in eight physiotherapy treatments and five evaluation sessions over a 6-week period. Potential participants were excluded if they: 1) had experienced a previous LAS in the 12 months preceding recruitment, 2) had ever suffered a foot or ankle fracture, 3) had residual signs or symptoms of a previous foot or ankle injury, 4) had any lacerations, wounds or other conditions affecting skin integrity at the treatment site, 5) presented any contraindications to cryotherapy treatment (such as peripheral vascular disease, Raynaud’s syndrome, cold urticaria, or cryoglobulinemia) or altered skin sensitivity. Potential participants were also excluded if the principal injury, as diagnosed in the baseline evaluation, was not to the lateral ankle ligaments, but rather to the distal tibiofibular syndesmosis (assessed with the squeeze test, the dorsiflexion-external rotation test, and palpation) [18–20]. For the purpose of this study, a moderate to high grade LAS diagnosis was confirmed with the presence of all of the followings: 1) an history of traumatic onset, 2) pain and limping (or incapacity) at walking, 3) visible edema on the lateral aspect of the ankle, and finally, 4) pain at palpation of the anterior talofibular ligament or the calcaneofibular ligament, and/or an augmented range of movement in at least one test among the anterior drawer, the anterior talofibular ligament stress test and the calcaneofibular ligament stress test. As there is no clear consensus on how to diagnose and classify LAS, these criteria were chosen to reflect current clinical guidelines [1, 5, 6, 21, 22], Potential participants presenting with positive Ottawa ankle rules who had not undergone radiography evaluation were sent for medical examination in order to exclude possible fractures [23]. The sectorial health sciences research ethics committee of *Université Laval* approved this study (#2015–053).

### Study design

**Fig. 1:**
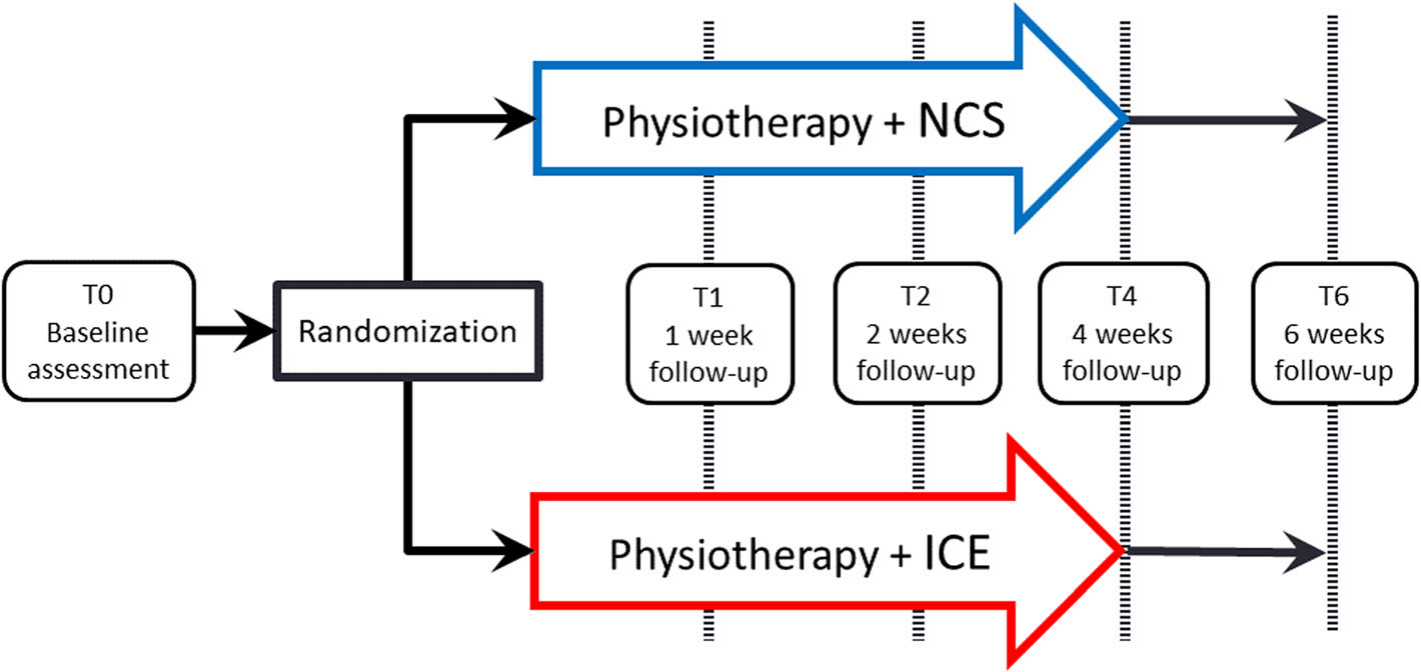
Study Design. NCS: neurocryostimulation

A single-blind, parallel group randomized clinical trial with blinded evaluator was realized (see Fig. 1. Study Design). Following the baseline evaluation, participants were randomly assigned to either the NCS (experimental: standardized rehabilitation program + NCS) or the ice (comparison: same standardized rehabilitation program + ice application) group. Both groups received standardized physiotherapy treatments, consisting of eight treatments over a four-week period (three treatments per week for two weeks, followed by one treatment per week for two weeks). They were evaluated five times in total: at baseline (T0), one week (T1), two weeks (T2), four weeks (T4) and six weeks (T6). Sociodemographic data were collected at T0. Functional capacity (the primary outcome), pain, ankle dorsiflexion ROM and edema measurements were taken at each of the five evaluation sessions.

### Randomization and blinding

A randomization list, generated by an independent researcher not involved in data collection, was established prior to initiation of the study using a random number generator (block randomization; block size of 4, 6 or 8). Randomization was stratified according to sex (male/female). Allocation was concealed in sealed and opaque envelopes sequentially numbered. After validation of the eligibility criteria, the baseline assessment was immediately conducted and then an independent researcher opened the sealed envelope and informed the treating physiotherapist of the group assignment. Participants were unaware of the treatment provided to the participants in the other group, nor that the cryotherapy was the central element of this RCT. Before every evaluation session, participants were reminded not to share their group assignment with the evaluator. All these evaluations took place in a closed room, separate from the treating area of the physiotherapy clinic. To assess blinding effectiveness, the evaluator answered the following question at the week-6 evaluation: *“In your opinion, which intervention did this participant receive?”* The possible answers were: NCS (experimental group); ice application (comparison group); I have no idea.

### Outcome measures

The primary outcome was functional capacity, as measured by the Lower Extremity Functional Scale (LEFS). The LEFS is a self-administered questionnaire consisting of 20 items [24]. It has been validated in individuals with lateral ankle sprain [25], and its reliability, construct validity and responsiveness have all been demonstrated [24–27]. The validated French Canadian version of the questionnaire was used for the current study [28, 29]. The minimal detectable change (MDC) and the minimal clinically important difference (MCID) are both 6 points [27].

Secondary dependent variables included pain intensity, ankle edema, and dorsiflexion ROM. Two visual analog scales (VAS) were filled to evaluate perceived pain intensity at rest and during usual activities in the last 48 h (10 cm scale varying from 0 [“no pain”] to 10 [“worst pain imaginable”]) [30]. The reliability of this scale has been demonstrated, and its MCID is 1.3 cm in individuals with acute pain [31]. The Figure of Eight method measures ankle size and edema using precise anatomical landmarks, and has been found to be reliable and valid in populations with ankle sprains or other injuries of the ankle or foot [32–34]. The minimal detectable change (MDC_95%_) is 0.96 cm for swollen ankles [35]. Finally, the employed dorsiflexion range of motion (ROM) measurement, the Weight-Bearing Lunge Test (WBLT), is standardized, simple, fast and reliable. The distance between the big toe and a wall is measured in a forward lunge in weight-bearing (with the knee touching the wall). Its MDC has been established at 1.6 cm (inter-evaluator) or 1.9 cm (intra-evaluator) [36].

### Interventions

Both groups received the same rehabilitation program. The only between-group difference was the application of ice in the comparison group and the use of NCS in the experimental group. Three physiotherapists, accustomed to working with individuals with acute ankle injuries, administered the treatments in an outpatient physiotherapy clinic on the university campus. All three physiotherapists treated participants within both allocation groups. They were properly trained in the study protocol and rehabilitation program, and they practiced the cryotherapy procedures often enough to be at ease and effective before the onset of the study. While they were asked to respect their own clinical judgment for the elaboration of their treatment plan, they were required to prescribe and teach stretching, strengthening and balance exercises and to perform joint mobilizations. They were also advised to use a compression and support modality such as a laced brace, taping or elastic bandaging. On the contrary, they were not permitted to use ultrasound, electrotherapy (such as transcutaneous electrical nerve stimulation or interferential current), heat application or dry needling modalities. This rehabilitation program follows current clinical guidelines [1, 5, 6, 37]. In-clinic sessions therefore consisted mainly of education on the injury and the healing process, teaching and practicing exercises, joint mobilization and soft tissue techniques, and management of compression and support methods. The parameters and details of each of these interventions were adjusted at the physiotherapist’s discretion according to each participant’s condition and progress.

In both groups, cryotherapy treatments were administered at the end of each physiotherapy session. For the NCS group, NCS was applied following the manufacturer’s instructions (Cryofos Medical GmbH, Germany), which is similar to application methods used in other studies [16, 17]. Slow sweeping motions of the medical gun were used over zones approximately the size of a credit card, for a maximum of 2 min, until a skin temperature of 4° Celsius was reached. Skin temperature was measured constantly by a thermometer contained within the medical gun, and the desired temperature of 4° Celsius was usually attained within 30 s of application. This procedure was applied to two distinct zones in order to cover the full surface area of the lateral ankle and was then repeated on the medial ankle and posterior to the knee, due to the presence of lymphatic ganglions at the latter site. For the ice group, the cryotherapy consisted of two bags of crushed ice (each approximately 25 × 25 cm) applied around the injured ankle in order to cover the joint entirely for 15 min, with the legs elevated. Skin temperature of the lateral treated ankle was measured before and immediately following each cryotherapy treatment in both groups, using the thermometer contained within the NCS medical gun.

### Sample size calculation

The required sample size was established at 36 subjects, 18 in each group, based on a standard deviation of 12.5 points and a MCID of 6.3 points on the primary outcome (LEFS questionnaire) [26] as well as a 15% loss to follow-up, using G*Power 3.1.7 software. Alpha and beta levels were set at α = 0.05, β = 0.80, with effect sizes of 0.80.

### Statistical analyses

Baseline sociodemographic data were compared between the groups using independent t-tests and chi-squared tests. Normality of the distributions was verified for each of the variables. Nonparametric Analysis for Longitudinal Data (nparLD Package 2.1, R-software, v.3.3.3) for repeated measures were used since distributions were normal at baseline (as there was a relatively wide range of scores for every single outcome) and gamma at the latest assessment times (as most participants improved close to optimal values). The nparLD is a procedure that manages a change of distribution between groups and measurement times [38]. A two-way (2-Groups [NCS, ice] × 5-Time [T0, T1, T2, T4 and T6]) nparLD was used to compare NCS and ice effects on each of the dependent variables. Intention-to-treat procedures were followed, and on the rare occasions that data were missing, the result from the previous evaluation was used (Last-Observation-carried-Forward method). The α criterion was always set at 5%.

## Results

**Fig. 2:**
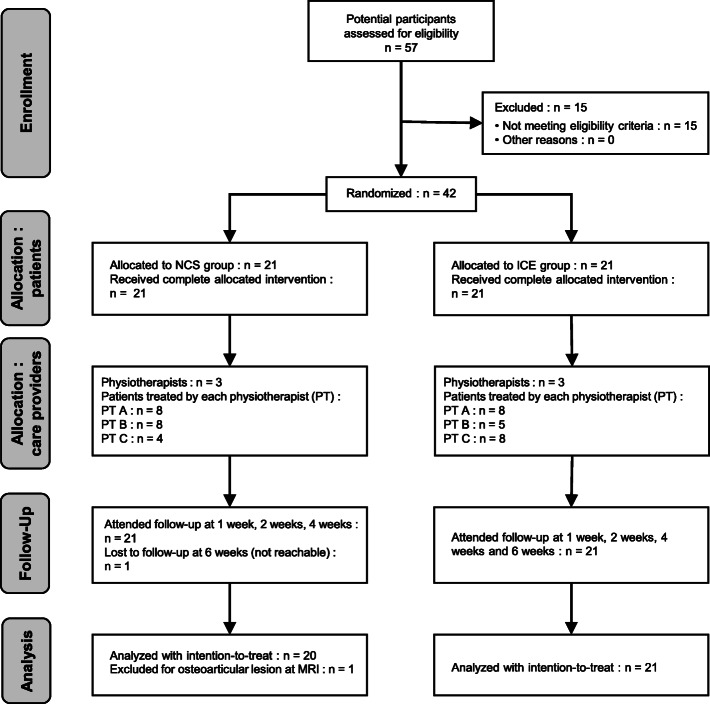
Flowchart of Participants. MRI: magnetic resonance imaging; NCS: neurocryostimulation; PT: physiotherapist

Fifty-seven potential participants were evaluated between June 2015 and October 2017. Of these, 42 met the inclusion criteria and were included in the study (see Fig. 2. Flowchart of Participants). Fifteen were excluded at this point for the following reasons: grade 1 sprain (neither pain nor limping in walking, *n* = 10), tibio-fibular syndesmosis (*n =* 3) or mid-foot (*n =* 1) as the primary site of injury, and presence of chronic knee pain limiting daily activities (*n =* 1). One participant, who was included in the study and randomized to the NCS group, still presented with major functional limitations at 6 weeks and was found to have ankle intra-articular osteochondral lesions on magnetic resonance imaging (MRI). This participant was excluded from the final analyses. No adverse event was reported during the course of the study among participants.

Average delays between the occurrence of the injury and the initial evaluation, and between the injury and the first treatment, were 2.2 (standard deviation [SD] 1.0) and 2.8 (SD 1.1) days, respectively. All subjects attended all eight treatments. Only one participant missed an evaluation (the 6-week follow-up, NCS group) and one other missed the edema and dorsiflexion ROM assessment at the 4-week and 6-week follow-ups (filled the LEFS and pain VAS remotely). Of the total outcome data, 13 measures were missing out of 1025, with only one missing for our primary outcome. The evaluator indicated that he remained unaware of group assignment for all participants throughout the study.

Participants’ baseline characteristics are presented in Table 1. No differences between the groups were found for all measured variables. In the NCS group, the average (mean of all eight sessions) decrease in skin temperature following cryotherapy was 23.7 °C (SD 4.6), compared to 15.3 °C (SD 5.0) in the ice group. Average (mean of all eight sessions) post-cryotherapy skin temperatures reached were 8.4 °C (SD 4.1) in the NCS group, and 16.9 °C (SD 5.0) in the ice group.

**Table 1:** Participants’ baseline characteristics

		NCS group (***n*** = 20)	Ice group (***n*** = 21)
Age, years, X̅ ± SD		26.9 ± 9.1	28.3 ± 9.8
Gender, # (% of allocation group)	Female	10 (50%)	10 (48%)
	Male	10 (50%)	11 (52%)
Weight, kg, X̅ ± SD		73.8 ± 12.9	72.1 ± 14.4
Height, cm, X̅ ± SD		172.4 ± 9.3	171.9 ± 9.4
Body mass index, kg/m^2^, X̅ ± SD		24.8 ± 3.8	24.5 ± 5.6
Number of previous ankle sprains, X̅ ± SD	Same ankle	0.9 ± 1.4	1.0 ± 1.4
	Any ankle	1.9 ± 2.8	1.5 ± 2.1
Physical activity, mean hours/week last 12 months, X̅ ± SD		10.6 ± 8.2	7.1 ± 4.5
Days from injury to initial assessment, X̅ ± SD		2.4 ± 0.9	2.0 ± 1.1
Days from injury to 1st treatment, X̅ ± SD		3.0 ± 1.1	2.5 ± 1.1

*NCS* neurocryostimulation, *SD* standard deviation, *X̅* mean. There was no statistical difference between the two groups for all variables (P ≥ 0.05; independent t-tests or Chi-squared tests).

**Fig. 3:**
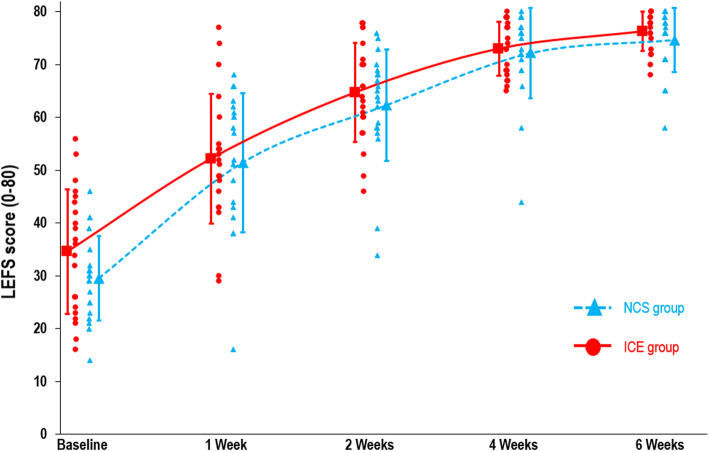
LEFS Scores. Lower Extremity Functional Scale (LEFS) mean scores (larger markers) for NCS group (*n* = 20) and ice group (*n* = 21), and individual scores (smaller markers). Higher scores indicate better function. Error bars show standard deviation (SD) of the means. NCS: neurocryostimulation

**Fig. 4:**
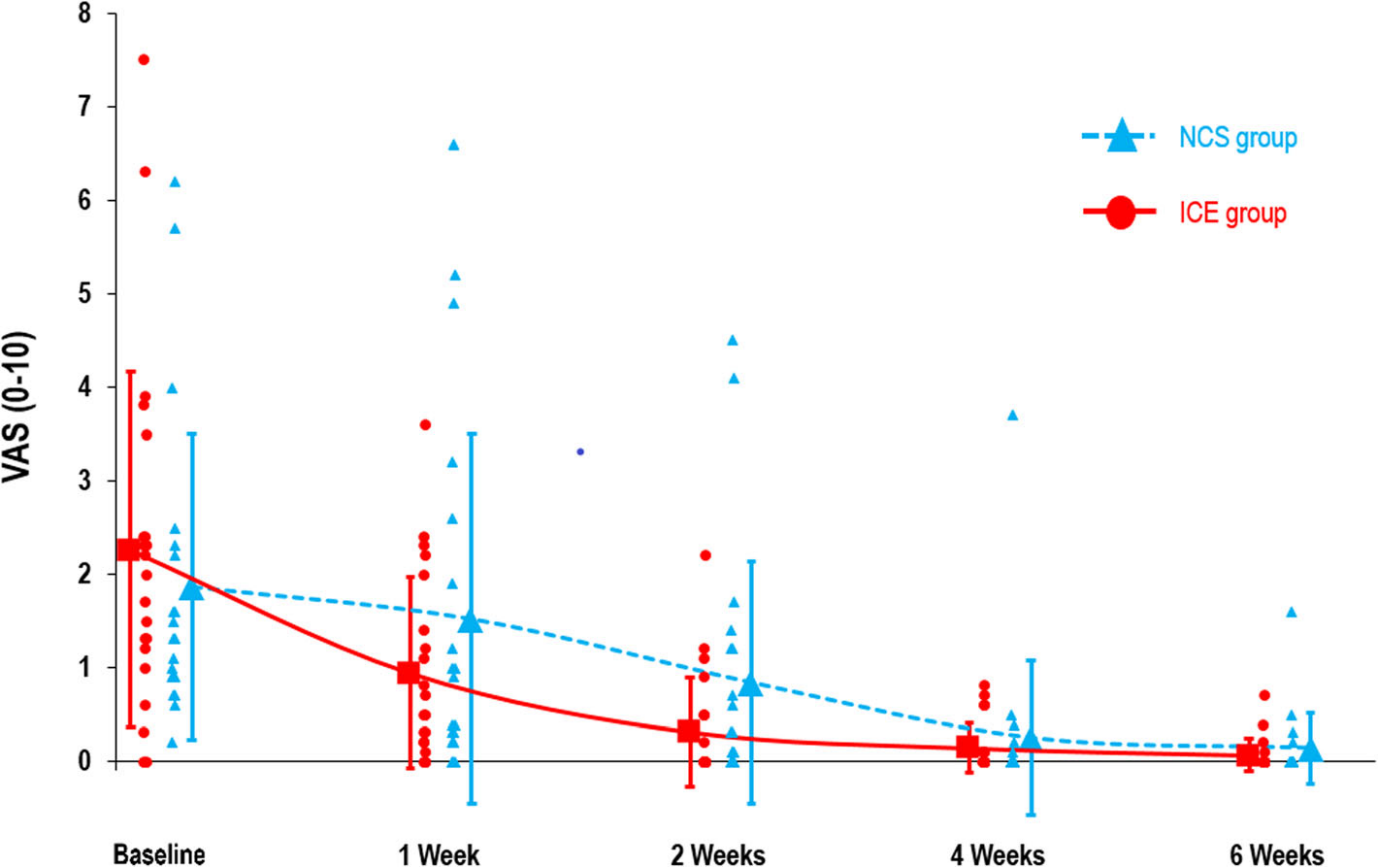
Pain at Rest Scores. Pain intensity at rest during the last 48 h. Data show mean scores (larger markers) for NCS group (*n =* 20) and ice group (*n =* 21), and individual scores (smaller markers). Error bars show standard deviation (SD) of the means. NCS: neurocryostimulation; VAS: visual analog scale

**Fig. 5:**
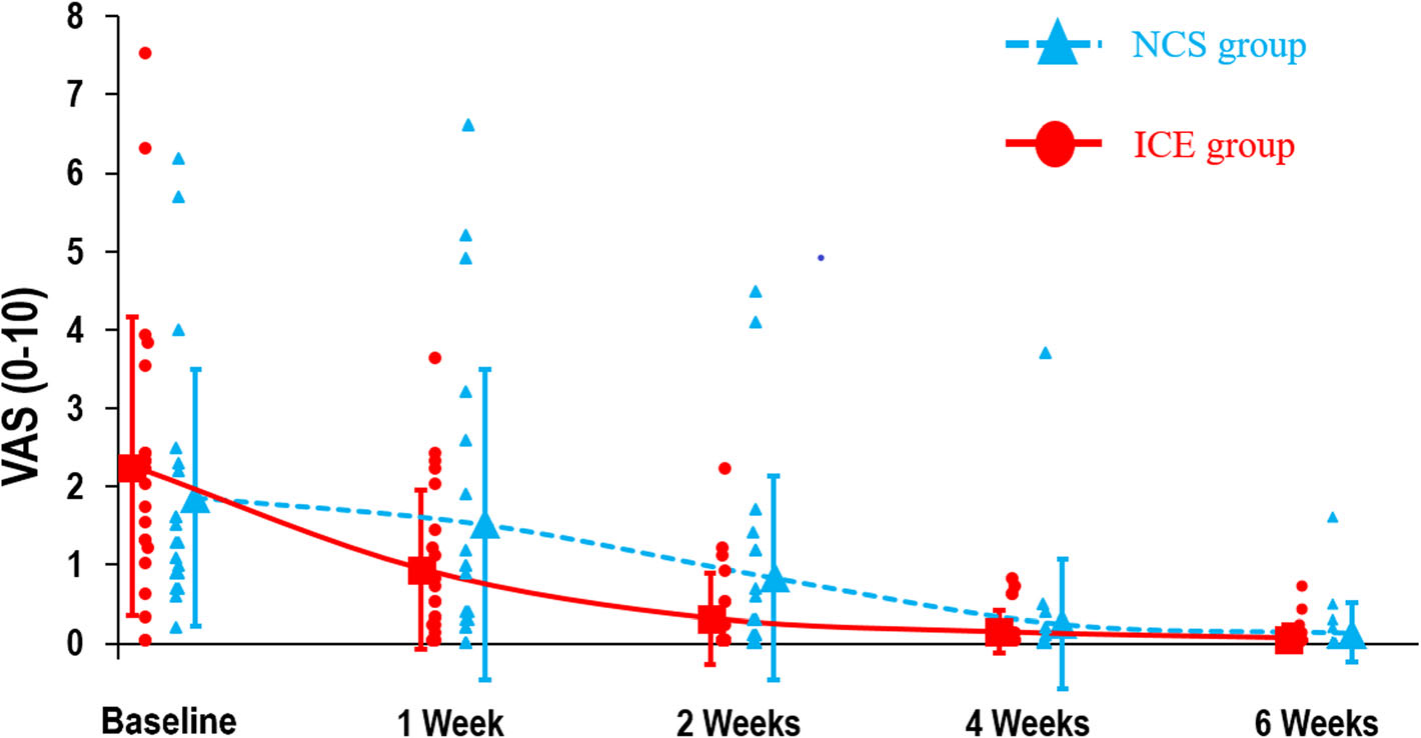
Pain during Usual Activities Scores. Pain intensity during usual activities for the last 48 h. Data show mean scores (larger markers) for NCS group (*n =* 20) and ice group (*n =* 21), and individual scores (smaller markers). Error bars show standard deviation (SD) of the means. NCS: neurocryostimulation; VAS: visual analog scale

Table 2 presents the *p*-values of the ANOVA-type analysis (nparLD). For the primary outcome, the LEFS, no group x time interaction was found (*p =* 0.73). Rapid improvement was observed in both groups, particularly during the first two weeks of treatment, with a large time effect (*p* <  0.001). In both the NCS and ice groups, LEFS mean score change was greater than the MCID between T0 and T1, T1 and T2, and T2 and T4. No group x time interaction was demonstrated for the secondary outcomes of pain intensity at rest and during usual activities (*p* = 0.06 and 0.65, respectively), dorsiflexion ROM (*p* = 0.77) and edema (*p* = 0.24), and substantial time effects were observed for all of these variables (*p <*  0.001). Table 3 presents the marginal estimated means by group at all measure times and Table 4 presents scores’ changes over time, for all outcomes. Figures 3, 4 and 5 shows graphically the evolution by group for the LEFS, pain at rest and pain during usual activities mean scores, respectively.

**Table 2:** Results (*p*-values) of ANOVA-type analysis (nparLD) for the intention-to-treat analysis

	Group effect	Time effect	Group X Time interaction
Functional capacity (LEFS)	0.404	< 0.0001	0.727
Pain at rest	0.390	< 0.0001	0.057
Pain during usual activities	0.995	< 0.0001	0.648
Oedema (Figure-of-8)	0.563	< 0.0001	0.242
Dorsiflexion ROM (WBLT)	0.408	< 0.0001	0.766

*LEFS* Lower Extremity Functional Scale, *nparLD* non-parametric longitudinal data, *ROM* range of motion, *WBLT* Weight Bearing Lunge Test.

**Table 3:** Group marginal estimated means for all outcomes. Data expressed as mean ± standard error of the mean (SEM)

	NCS (experimental group, ***n*** = 20)	Ice (comparison group, ***n*** = 21)
**Functional capacity (LEFS scores, 0–80)**		
Baseline	29.6 ± 1.7	34.7 ± 2.5
1 week	51.5 ± 2.9	52.2 ± 2.6
2 weeks	62.4 ± 2.3	64.7 ± 2.0
4 weeks	72.2 ± 1.9	73.0 ± 1.1
6 weeks	74.7 ± 1.3	76.3 ± 0.8
**Pain at rest (VAS, 0–10)**		
Baseline	1.9 ± 0.4	2.2 ± 0.4
1 week	1.5 ± 0.4	0.9 ± 0.2
2 weeks	0.8 ± 0.3	0.3 ± 0.1
4 weeks	0.2 ± 0.2	0.1 ± 0.1
6 weeks	0.1 ± 0.1	0.1 ± 0.0
**Pain during usual activities (VAS, 0–10)**		
Baseline	4.5 ± 0.5	4.6 ± 0.4
1 week	2.4 ± 0.4	2.3 ± 0.4
2 weeks	1.7 ± 0.4	1.2 ± 0.2
4 weeks	0.7 ± 0.3	0.5 ± 0.2
6 weeks	0.4 ± 0.2	0.2 ± 0.1
**Oedema (Figure-of-8, cm)**		
Baseline	53.7 ± 0.7	52.7 ± 0.8
1 week	53.0 ± 0.7	52.0 ± 0.8
2 weeks	52.6 ± 0.7	52.1 ± 0.8
4 weeks	52.4 ± 0.6	51.7 ± 0.7
6 weeks	52.5 ± 0.7	51.9 ± 0.8
**Dorsiflexion ROM (WBLT, cm)**		
Baseline	5.5 ± 0.8	5.9 ± 1.0
1 week	7.9 ± 0.9	9.4 ± 0.5
2 weeks	9.6 ± 0.7	10.6 ± 0.5
4 weeks	10.7 ± 0.5	11.7 ± 0.5
6 weeks	11.2 ± 0.5	11.7 ± 0.6

*LEFS* Lower Extremity Functional Scale, *NCS* neurocryostimulation, *ROM* range of motion, *VAS* visual analog scale, *WBLT* Weight-Bearing Lunge Test.

**Table 4:** Outcomes changes over time compared to baseline values throughout treatment (marginal estimated mean improvements), by group

	Mean score change (95% CI)		Time main effect (RTE)^**a**^ (***n*** = 41)
	NCS (experimental group, ***n*** = 20)	Ice (comparison group, ***n*** = 21)	
**Functional capacity (LEFS scores, 0–80)**			
1 week	22.0 (13.6 to 30.3)	17.6 (10.2 to 25.0)	0.340
2 weeks	32.8 (26.0 to 39.6)	30.1 (22.4 to 37.7)	0.514
4 weeks	42.7 (37.2 to 48.1)	38.3 (31.1 to 45.6)	0.716
6 weeks	45.2 (40.3 to 50.0)	41.6 (33.5 to 49.7)	0.800
**Pain at rest (VAS, 0–10)**			
1 week	− 0.4 (+ 0.8 to − 1.5)	−1.3 (− 0.6 to − 2.1)	0.375
2 weeks	−1.1 (+ 0.2 to − 2.3)	−1.9 (− 0.8 to −3.0)	0.534
4 weeks	− 1.6 (− 0.3 to − 2.9)	− 2.1 (− 0.8 to − 3.4)	0.668
6 weeks	− 1.8 (− 0.6 to − 2.9)	− 2.2 (− 0.9 to − 3.4)	0.705
**Pain during usual activities (VAS, 0–10)**			
1 week	− 2.1 (− 0.6 to − 3.6)	− 2.3 (− 1.3 to − 3.3)	0.382
2 weeks	−2.8 (− 1.5 to − 4.2)	−3.4 (− 2.4 to − 4.4)	0.490
4 weeks	−3.8 (− 2.4 to − 5.2)	−4.1 (− 2.9 to − 5.3)	0.682
6 weeks	−4.1 (− 2.7 to − 5.6)	−4.4 (− 3.2 to − 5.6)	0.774
**Oedema (Figure-of-8, cm)**			
1 week	−0.69 (− 0.05 to − 1.34)	−0.64 (+ 0.11 to − 1.38)	0.496
2 weeks	− 1.06 (− 0.29 to − 1.83)	−0.56 (+ 0.28 to − 1.40)	0.508
4 weeks	− 1.24 (− 0.29 to − 2.18)	−0.93 (− 0.19 to − 1.67)	0.532
6 weeks	−1.15 (− 0.20 to − 2.09)	−0.76 (− 0.05 to − 1.47)	0.519
**Dorsiflexion ROM (WBLT, cm)**			
1 week	2.4 (− 0.1 to 4.9)	3.6 (1.1 to 6.0)	0.414
2 weeks	4.1 (1.9 to 6.3)	4.8 (2.2 to 7.4)	0.550
4 weeks	5.2 (3.0 to 7.4)	5.9 (3.2 to 8.6)	0.643
6 weeks	5.7 (3.4 to 8.0)	5.9 (3.1 to 8.7)	0.668

^a^Relative treatment effect (nparLD analysis), for appreciation of the time main effect. Corresponds to an effect size of the time. Values range [0, 1], relative to the null hypothesis (H0) expected value (0,5)

*LEFS* Lower Extremity Functional Scale, *NCS* neurocryostimulation, *ROM* range of motion, *RTE* relative treatment effect, *VAS* visual analog scale, *WBLT* Weight-Bearing Lunge Test.

## Discussion

This RCT compared the effect of two cryotherapy interventions, namely neurocryostimulation and traditional ice application, on recovery in individuals suffering from acute LAS receiving physiotherapy treatments. Results indicated no difference between ice application and NCS throughout the 6-week follow-up period for any of the outcomes. Analyses demonstrated large time effects for all studied variables, as all participants improved rapidly regardless of treatment modality. There are several possible explanations for the comparable results seen between the groups: 1) the interventions are equally effective, 2) neither of the interventions is more effective than the mere passage of time, or 3) possible between-group differences were hidden by a stronger effect of the global physiotherapy treatment, or by the participants’ rapid improvement, which is typical of this type of injury. Potential differences between NCS and ice application effects could thus be better detected in populations with conditions or injuries that typically progress more slowly.

Few clinical studies measuring the effect of NCS in populations presenting with musculoskeletal injuries have been published in peer-reviewed journals, and none has treated ankle sprains specifically. When studying the effect of NCS in the elderly, Chatap et al. concluded that NCS decreases acute and chronic pain of various etiologies;[16 ] however, they did not have a control or comparison group. In contrast, in a pilot study, Richer et al. failed to find differences when comparing the effects of NCS combined with manual therapy to those of manual therapy alone on pain, function and pain-free grip strength in patients with chronic lateral epicondylitis [39]. Similarly, in a RCT, Demoulin et al. did not find any between-group differences when comparing the effects of NCS and two other types of cryotherapy (gel packs and c*ryocuffs*) on pain, knee mobility and edema seven days after total knee replacement surgery[17]. Of note, the NCS treatment protocol in Demoulin et al.’s study resulted in cooling of the skin to an average temperature of 14 °C, which is warmer than the temperatures recommended to expect benefits from cryotherapy[40, 41].

This methodological limitation regarding the level of cooling is one important reason why the use of cold in injury care is often questioned [11, 12]. Cryotherapy has been proposed to control pain by slowing nerve conduction and to decrease secondary ischemic or enzymatic tissue death[42]. Demonstrated effects of cryotherapy include significant cutaneous analgesia below 13.6 °C, a 10% decrease in nerve conduction below 12.5 °C, and a 50% decrease in local metabolism below 11 °C [40, 41]. In numerous studies looking at the effects of cryotherapy, those goal temperatures have not been reached, [13] contributing to the persistent difficulty in clarifying the clinical usefulness of cryotherapy in health care. In fact, the achieved temperature depends on many factors, such as the cryotherapy method used, the duration of application, the initial skin temperature and the thickness of the subcutaneous fatty tissue, among others. In our study, the skin temperature attained following the application of ice did not reach the mentioned thresholds, while it did reach these thresholds following NCS.

Mourot et al. demonstrated that after NCS, the rate of rewarming during the first minutes is quicker than after ice application, and that skin temperature is no longer significantly different between groups after one [15] or six minutes [14]. This could suggest that the level of deep tissue induced cooling would be similar between NCS and ice, and that the additional cooling induced by NCS would be limited to the superficial tissues. In terms of potential analgesic and metabolic slowing effects in deep injured tissues, such as ligaments in the case of sprains, NCS and ice therefore likely have similar effects, and the effects on the autonomic nervous system attributed to NCS, the so-called “thermal shock”, may result from a greater or quicker cooling of the surface structures only, and not of the deep structures.

Although there is no evidence of the clinical effectiveness of NCS, its physiological effects on circulatory parameters have been described. Mourot et al. observed rapid systemic cutaneous vasoconstriction and increased blood pressure in response to NCS application in healthy subjects,[14, 15] which are reactions comparable to those observed when evaluating the effect of hand immersion in cold water (cold pressor test )[43]; they did not, however, see those reactions with ice application [14, 15]. This response, referred to as thermal shock and involving the autonomic nervous system, is the basis of the hypothesis that NCS holds potential as a treatment modality. In the current study, rapid and significantly greater decreases in skin temperature were observed in the NCS group compared to the ice group, suggesting that a thermal shock was generally obtained in the former group. That did not, however, translate into the greater therapeutic benefits that were expected.

From a more practical point of view, the use of NCS requires a substantial investment of money at the time of purchase, in addition to the cost of periodic refilling of the gas cylinder and eventual maintenance of the unit. It is therefore more expensive than the use of ice, and also has the disadvantage of not being easily transportable for use at home, or on the sports field, for examples. There are smaller models of NCS devices designed to be transported, but those were not used in this study. NCS nevertheless holds a distinct advantage over ice application: with an application time of less than two minutes, it is much quicker for the patient than the ten, fifteen or even twenty minute application period recommended when using ice [13, 44].

When interpreting the results of this study, some limitations should be kept in mind, the main one being that there was no control group, i.e. a group not receiving cryotherapy of any kind. Including such a group would have made it possible to study the effects of both NCS and ice. This study also did not control for placebo effect, which would have required creating a treatment similar to NCS, but with no effects related to cooling of the skin; however, a credible sham NCS that would feel cold enough without actually lowering skin temperature significantly seemed impossible to put in place. Regarding treatment frequency, optimal NCS application parameters are unknown. Here, treatments were offered at a frequency of three times per week for the first two weeks, and once per week for the following two weeks. These parameters were chosen because they were considered a good compromise between the desire to administer numerous treatments in the very acute phase of the injury, and the logistical challenges that are intrinsic to delivering outpatient physiotherapy treatments, such as scheduling and travel time.

Although physiotherapists were given specific guidelines for choosing which interventions to use for the rehabilitation program, the exact parameters for interventions other than cryotherapy were neither imposed nor recorded. This is a potential source of variation between participants that could have affected the results. Also, the protocol for the ice group included elevation of the lower extremity, with ice applied all around the ankle with bags of crushed ice that also applied compression. The potential effects of ice cannot therefore be isolated from those of compression and elevation. Finally, the age of the participants, with a mean of 28 years (SD: 9), characterizes a relatively young sample of patients, which may be a limitation to the overall generalizability of the findings.

Conversely, one of the greatest strengths of this study was the ability to include participants quickly, as evidenced by the average of 2.8 days between the injury and the first treatment received. Another strength is the study design which made it possible to evaluate the progression of patients during the phase of intense metabolic and inflammatory activity (1-week and 2-weeks follow-ups). Also, the near-perfect retention rate is worth mentioning.

## Conclusion

The results of this RCT suggest that a more sophisticated and costly application of cryotherapy, namely neurocryostimulation, is no more effective than the traditional application of ice in improving functional recovery, pain, edema and ankle dorsiflexion ROM during the first six weeks of physiotherapy treatments in individuals with an acute lateral ankle sprain. Other RCTs would be useful to better evaluate the potential utility or superiority of this treatment modality by including different populations, injuries and pathologies, varying NCS application protocols, and including a proper control group not receiving any cryotherapy. Altogether, current evidence suggests that the only advantage of NCS compared to ice is the rapidity with which it is applied, which may be a key element to consider in a busy high-volume rehabilitation practice.

## Data Availability

The datasets used and/or analysed during the current study are available from the corresponding author on request.

## Abbreviations

CI: Confidence interval
LAS: Lateral ankle sprain
LEFS: Lower extremity functional scale
MCID: Minimal clinically important difference
MDC: Minimal detectable change
MRI: Magnetic resonance imaging
NCS: Neurocryostimulation
nparLD: Nonparametric analysis for longitudinal data
RCT: Randomized clinical trial
RICE: Rest, ice, compression, elevation
ROM: Range of motion
SD: Standard deviation
SEM: Standard error of measurement
VAS: Visual analog scale
WBLT: Weight-bearing lunge test
X̅: Mean

## References

[1] van den Bekerom MP, Kerkhoffs GM, McCollum GA, Calder JD, van Dijk CN (2013). Management of acute lateral ankle ligament injury in the athlete. Knee Surg Sports Traumatol Arthrosc.

[2] Medical M's (2005). Nursing & Allied Health Dictionary. 5th edition ed.

[3] Doherty C, Delahunt E, Caulfield B, Hertel J, Ryan J, Bleakley C (2014). The incidence and prevalence of ankle sprain injury: a systematic review and meta-analysis of prospective epidemiological studies. Sports Med.

[4] Verhagen EA, van Mechelen W, de Vente W (2000). The effect of preventive measures on the incidence of ankle sprains. Clin J Sport Med.

[5] Kerkhoffs GM, van den Bekerom M, Elders LA (2012). Diagnosis, treatment and prevention of ankle sprains: an evidence-based clinical guideline. Br J Sports Med.

[6] Kaminski TW, Hertel J, Amendola N (2013). National Athletic Trainers' association position statement: conservative management and prevention of ankle sprains in athletes. J Athl Train.

[7] van den Bekerom MP, Struijs PA, Blankevoort L, Welling L, van Dijk CN, Kerkhoffs GM (2012). What is the evidence for rest, ice, compression, and elevation therapy in the treatment of ankle sprains in adults?. J Athl Train.

[8] Vuurberg G, Hoorntje A, Wink LM, et al. Diagnosis, treatment and prevention of ankle sprains: update of an evidence-based clinical guideline. Br J Sports Med. 2018. 10.1136/bjsports-2017-098106 29514819

[9] Tiemstra JD (2012). Update on acute ankle sprains. Am Fam Physician.

[10] Chaudhry H, Simunovic N, Petrisor B (2015). Cochrane in CORR (R): surgical versus conservative treatment for acute injuries of the lateral ligament complex of the ankle in adults (review). Clin Orthop Relat Res.

[11] Doherty C, Bleakley C, Delahunt E, Holden S (2017). Treatment and prevention of acute and recurrent ankle sprain: an overview of systematic reviews with meta-analysis. Br J Sports Med.

[12] Bleakley C, McDonough S, MacAuley D (2004). The use of ice in the treatment of acute soft-tissue injury: a systematic review of randomized controlled trials. Am J Sports Med.

[13] MacAuley D (2001). Ice therapy: how good is the evidence?. Int J Sports Med.

[14] Mourot L, Cluzeau C, Regnard J (2007). Hyperbaric gaseous cryotherapy: effects on skin temperature and systemic vasoconstriction. Arch Phys Med Rehabil.

[15] Mourot L, Cluzeau C, Regnard J (2007). Physiological assessment of a gaseous cryotherapy device: thermal effects and changes in cardiovascular autonomic control. Ann Readapt Med Phys.

[16] Chatap G, De Sousa A, Giraud K, Vincent JP (2007). Acute pain in the elderly study G. pain in the elderly: prospective study of hyperbaric CO2 cryotherapy (neurocryostimulation). Joint Bone Spine.

[17] Demoulin C, Brouwers M, Darot S, Gillet P, Crielaard JM, Vanderthommen M (2012). Comparison of gaseous cryotherapy with more traditional forms of cryotherapy following total knee arthroplasty. Ann Phys Rehabil Med.

[18] Sman AD, Hiller CE, Refshauge KM (2013). Diagnostic accuracy of clinical tests for diagnosis of ankle syndesmosis injury: a systematic review. Br J Sports Med.

[19] Schwieterman B, Haas D, Columber K, Knupp D, Cook C (2013). Diagnostic accuracy of physical examination tests of the ankle/foot complex: a systematic review. Int J Sports Phys Therapy.

[20] Lau BC, Moore LK, Thuillier DU (2018). Evaluation and Management of Lateral Ankle Pain Following Injury. JBJS reviews.

[21] Lynch SA (2002). Assessment of the injured ankle in the athlete. J Athl Train.

[22] Wiebking U, Jagodzinski M, Pacha TO (2015). An accuracy evaluation of clinical, arthrometric, and stress-sonographic acute ankle instability examinations. Foot Ankle Surg.

[23] Stiell IG, Greenberg GH, McKnight RD, Nair RC, McDowell I, Worthington JR (1992). A study to develop clinical decision rules for the use of radiography in acute ankle injuries. Ann Emerg Med.

[24] Binkley J, Stratford P, Lott S, Riddle D (1999). The lower extremity functional scale (LEFS) scale development, measurement properties, and clinical application. Phys Ther.

[25] Alcock GK, Stratford PW. Validation of the lower extremity functional scale on athletic subjects with ankle sprains. Physiother Can. 2002;(Fall 2002):233–40.

[26] Pan S-L, Liang H-W, Hou W-H, Yeh T-S (2014). Responsiveness of SF-36 and Lower Extremity Functional Scale for assessing outcomes in traumatic injuries of lower extremities. Injury.

[27] Mehta SP, Fulton A, Quach C, Thistle M, Toledo C, Evans NA (2016). Measurement properties of the lower extremity functional scale: a systematic review. J Orthop Sports Phys Ther.

[28] Rene F, Casimiro L, Tremblay M (2011). [Fiabilité test retest et validité de construit de la version française de L'Échelle fonctionnelle des membres inférieurs (ÉFMI), partie II.]. Physiotherapy Canada. Physiotherapie Canada Spring.

[29] Rene F, Casimiro L, Tremblay M (2011). [Une version canadienne française du Lower Extremity Functional Scale (LEFS): L'Échelle fonctionnelle des membres inférieurs (ÉFMI), partie I.]. Physiotherapy Canada. Physiotherapie Canada Spring.

[30] Katz J, Melzack R (1999). Measurement of pain. Surg Clin North Am.

[31] Gallagher EJ, Liebman M, Bijur PE (2001). Prospective validation of clinically important changes in pain severity measured on a visual analog scale. Ann Emerg Med.

[32] Mawdsley RH, Hoy DK, Erwin PM (2000). Criterion-related validity of the figure-of-eight method of measuring ankle edema. J Orthop Sports Phys Ther.

[33] Peterson EJ, Irish SM, Lyons CL (1999). Reliability of Water Volumetry and the Figure of Eight Method on Subjects With Ankle Joint Swelling. J Orthopaedic Sports Physical Therapy.

[34] Tatro-Adams D, McGann SF, Carbone W (1995). Reliability of the figure-of-eight method of ankle measurement. J Orthop Sports Phys Ther.

[35] Rohner-Spengler M, Mannion A, Babst R (2007). Reliability and minimal detectable change for the figure-of-eight-20 method of measurement of ankle edema. J Orthop Sports Phys Ther.

[36] Powden CJ, Hoch JM, Hoch MC. Reliability and minimal detectable change of the weight-bearing lunge test: A systematic review. Man Ther. 2015. 10.1016/j.math.2015.01.004 25704110

[37] Petersen W, Rembitzki IV, Koppenburg AG (2013). Treatment of acute ankle ligament injuries: a systematic review. Arch Orthop Trauma Surg.

[38] Noguchi K, Gel Y, Brunner E, Konietschke F (2012). nparLD: an R software package for the nonparametric analysis of longitudinal data in factorial experiments. J Stat Softw.

[39] Richer N, Marchand AA, Descarreaux M (2017). Management of Chronic Lateral Epicondylitis with Manual Therapy and Local Cryostimulation: a pilot study. J Chiropractic Med.

[40] McMeeken J, Lewis MM, Cocks S (1984). Effects of cooling with simulated ice on skin temperature and nerve conduction velocity. Aust J Physiother.

[41] Zachariassen KE (1991). Hypothermia and cellular physiology. Arctic Med Res.

[42] Hubbard TJ, Denegar CR (2004). Does Cryotherapy improve outcomes with soft tissue injury?. J Athl Train.

[43] Pouwels S, Van Genderen ME, Kreeftenberg HG (2019). Utility of the cold pressor test to predict future cardiovascular events. Expert Rev Cardiovasc Ther.

[44] Bleakley C, McDonough S, MacAuley D (2006). Cryotherapy for acute ankle sprains: a randomised controlled study of two different icing protocols. Br J Sports Med.

